# Self‐Healing Hydrogels and Cryogels from Biodegradable Polyurethane Nanoparticle Crosslinked Chitosan

**DOI:** 10.1002/advs.201901388

**Published:** 2019-11-11

**Authors:** Tzu‐Wei Lin, Shan‐hui Hsu

**Affiliations:** ^1^ Institute of Polymer Science and Engineering National Taiwan University Taipei 10617 Taiwan ROC

**Keywords:** chitosan, crosslinkers, cryogels, polyurethane nanoparticles, self‐healing hydrogels

## Abstract

Hydrogels are widely used in tissue engineering owing to their high water retention and soft characteristics. It remains a challenge to develop hydrogels with tunable degradation rates, proper environmental responsiveness, and injectability. In this study, biodegradable difunctional polyurethane (DFPU) nanoparticle dispersions are synthesized from an eco‐friendly waterborne process involving the use of glyoxal. Such DFPU is used to crosslink chitosan (CS). Schiff base linkages between DFPU and CS successfully produce self‐healing hydrogels at room temperature. Moreover, cryogels are generated after being frozen at −20 °C. These gels are found to be sensitive to low pH and amine‐containing molecules owing to the property of Schiff bases. Furthermore, the degradation rates can be adjusted by the type of the component oligodiols in DFPU. Rheological evaluation verifies the excellent self‐healing properties (≈100% recovery after damage). Both the self‐healing gels and cryogels are injectable (through 26‐gauge and 18‐gauge needles, respectively) and biocompatible. Rat implantation at 14 d shows the low immune responses of cryogels. The functionalized biodegradable polyurethane nanoparticles represent a new platform of crosslinkers for biomacromolecules such as chitosan through the dynamic Schiff reaction that may give rise to a wide variety of self‐healing gels and cryogels for biomedical applications.

## Graphical abstract

A new biodegradable waterborne difunctional polyurethane (DFPU) crosslinker is obtained by modifying waterborne polyurethane (PU) with glyoxal. Adding the crosslinker to glycol chitosan (CS) produces CS‐PU self‐healing hydrogel at room temperature and generates CS‐PU cryogel after being temporally frozen at −20 °C. Both gels are featured with injectability (26‐gauge and 18‐gauge needles, respectively), multiresponsiveness, tunable degradation rates, good cell proliferation, and biocompatibility.

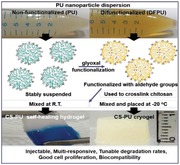

## 1 Introduction

Hydrogels are polymers with high water retention, soft characteristics, and good biocompatibility, which gain wide applications in biomedical fields. Recent attention has been paid to environmentally responsive hydrogels, injectable hydrogels, self‐healing hydrogels, and cryogels as potential biomedical materials owing to their excellent functionality.^1^ Environmentally responsive hydrogels are also known as smart hydrogels, which can change their chemical properties or physical structures in response to environmental stimuli and are often used in drug release and sensors.^2^ Injectable hydrogels have in situ formability which allows surgical operation in a minimally invasive way. Therefore, they hold promises in drug delivery, cell encapsulation, and tissue engineering.^3^ Meanwhile, self‐healing hydrogels are inspired by the self‐healing ability in biology.^4^ Materials with the ability of self‐healing have potential biomedical applications, such as drug release, capillary network construction, and hemostasis.^5^ Cryogels have attractive features of interconnected macropores' structure that allow the transport of nutrients and wastes through the cryogels.^6^ Cryogels are often used in chromatographic materials, efficient carriers for the immobilization of biomolecules and cells, and matrices for cell separation, cell culture, and tissue padding.^7^

Chitosan is a polycationic biopolymer that is naturally derived and is one of the important environmentally friendly renewable materials obtained by deacetylation of chitin.^8^ When the degree of deacetylation of chitin reaches about 50%, it becomes soluble in acidic condition and is named chitosan.[qv: 8b] Chitosan is generally nontoxic, biocompatible, biodegradable, and bacteriostatic, and has been employed for many pharmaceutical and medical applications including orthopedic/periodontal devices, tissue engineering, wound healing, and drug/gene delivery.^9^ However, chitosan can only be dissolved in water under acidic conditions, limiting its use as a living cell and tissue matrix.[qv: 5a] Therefore, the water‐soluble derivatives of chitosan such as glycol chitosan and N‐carboxyethyl chitosan have been developed that can be prepared as hydrogels.[qv: 5a,10]

Self‐healing hydrogels based on chitosan derivatives have attracted much recent attention.[qv: 5a,11] For the purpose, various crosslinkers have been synthesized and used. Among them, functionalized polyethylene glycol (PEG) crosslinkers are the most popular choices,[qv: 5a] for example, difunctional polyethylene glycol (DFPEG). DFPEG has good solubility, biocompatibility, and reacts with chitosan to form Schiff base (also known as imine, —N=CH—). The dynamic covalent bond in DFPEG crosslinked chitosan has the ability to deform and reform due to the Schiff base instability, and such structurally dynamic hydrogels can respond to many chemical and biological stimuli by liquefaction of the hydrogels.^12^ However, the hydrogel formed by DFPEG or multifunctionalized PEG may be dissolved rather rapidly in vivo.^13^ Besides, the soluble PEG cannot be decomposed in vivo.^14^ Therefore, biodegradable crosslinkers which have turnable degradation rates are demanded in the field. Meanwhile, Schiff base crosslinkers such as glutaraldehyde and dextran oxide are also used to prepare chitosan cryogels.^15^ As a crosslinker, DFPEG is not reported for cryogels, and dextran oxide is not reported for self‐healing hydrogels. Issues regarding if self‐healing hydrogels and cryogel can be made of the same main chain and crosslinker are rarely discussed.

Recent attention has been paid to nanoparticles as crosslinker because of their potential benefits. Incorporation of inorganic nanoparticles has been demonstrated to reinforce the hydrogel matrix, resulting in stronger nanocomposite gels.^16^ Through specific interactions with the hydrogel polymer chains, nanoparticles can effectively contribute to the polymer network elasticity and thereby increase the mechanical strength of the hydrogels.^17^ Furthermore, dynamic hydrogels with intrinsic self‐healing capabilities can result if the polymer–particle interfacial crosslinks are reversible.^18^ Moreover, nanoparticles can introduce a variety functionalities of inorganic materials to the hydrogel, such as electronic conductivity and magnetic response.^19^ Despite the great achievements of inorganic nanoparticle crosslinkers, the poor biodegradability and biocompatibility may limit their biomedical applications.

Based on the literature, biodegradable crosslinkers for chitosan self‐healing hydrogels or cryogels are still limited, and thus highly demanded. Here, we report an effective and novel crosslinker type of Schiff base from eco‐friendly waterborne polyurethane in nanoparticle (NP) form. By adjusting the reaction temperature, self‐healing hydrogels or cryogels with multiresponsiveness and injectability can be easily prepared. Novel self‐healing hydrogels and cryogels are injectable through 26‐gauge and 18‐gauge needles, respectively. Moreover, the soft segments of the developed crosslinker can be altered to obtain biodegradable hydrogels and cryogels with tunable degradation rates.

## 2 Results

### 2.1 Synthesis and Characterization of Difunctional Polyurethane (DFPU) Crosslinkers

**Figure 1:**
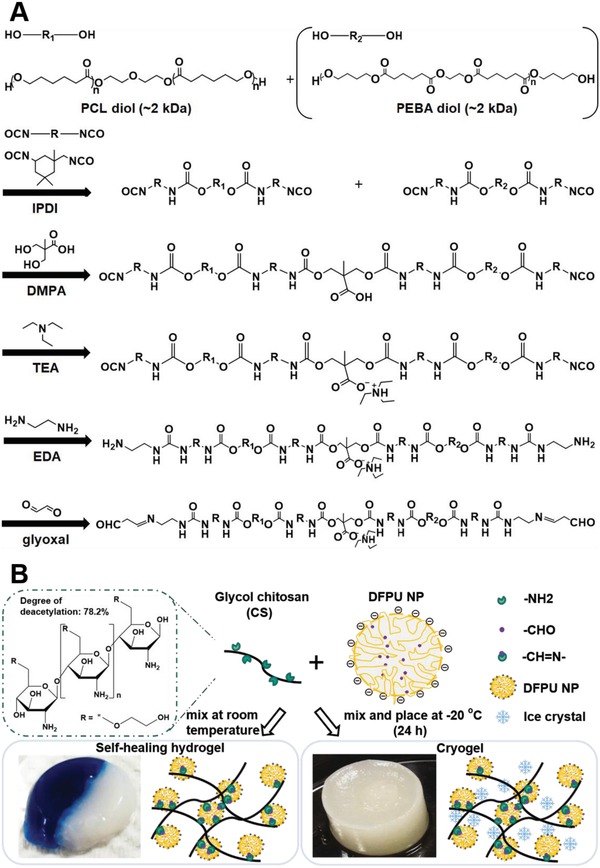
A) The synthetic route of novel DFPU crosslinkers. The oligodiol employed was PCL diol alone, or a combination of PCL diol with another oligodiol (such as PEBA diol). B) Schematic representation of the simple process to form self‐healing hydrogel or cryogel.

New difunctional polyurethane crosslinkers with tunable degradation rates were synthesized, as shown in **Figure** **1**A. The synthesis was based on a green, waterborne procedure. Two types of difunctional polyurethane crosslinkers were synthesized in this study. The major one was abbreviated as DFPU. The oligodiol employed to synthesize DFPU was polycaprolactone diol (PCL diol, Mn 2000 Da). The second difunctional polyurethane crosslinker with a different soft segment and faster degradation rate was abbreviated as DFPU'. The oligodiol employed to synthesize DFPU' was a combination of PCL diol and poly(1,4‐butylene adipate) diol (PEBA diol, Mn 2000 Da) in 2:3 mass ratio. 10 g of oligodiol and 3 g of isophorone diisocyanate (IPDI) were added in a glass vessel with the catalyst Sn(Oct)2 and reacted for 3 h under a nitrogen atmosphere at 75 °C. After that, 4.5 g of methyl ethyl ketone (MEK) and 0.6699 g of 2,2‐bis(hydroxymethyl) propionic acid (DMPA) were added and reacted for 1 h. 0.505 g of triethylamine (TEA) was added at 50 °C for 30 min. 0.21 g of ethylenediamine (EDA) and 36 mL of distilled water were added to the vessel under vigorous stirring for 1 h. Finally, 0.5075 g of glyoxal was added to the vessel and reacted for 30 min. The stoichiometric ratio of oligodiols/IPDI/DMPA/TEA/EDA/glyoxal was 1:2.7:1:1:0.7:0.7. As displayed in Figure 1B, the mixture of DFPU and glycol chitosan (CS) formed self‐healing hydrogel at room temperature (abbreviated as CS‐PU hydrogel) and formed cryogel after being frozen at −20 °C (abbreviated as CS‐PU cryogel).

**Figure 2:**
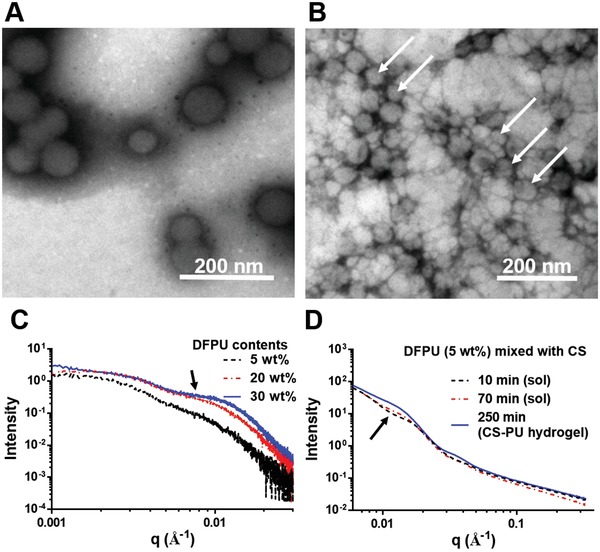
Characterization of DFPU by TEM and SAXS. A) TEM images for DFPU NPs alone. The size was in the range of 30–50 nm. B) TEM images for DFPU NPs mixed with glycol chitosan. The shape of DFPU nanoparticles changes when mixing with CS. C) SAXS profiles (measured at 37 °C) for DFPU dispersions of various solid contents (5–30 wt%). D) SAXS profiles for DFPU (5 wt%) mixed with twice the volume of glycol chitosan (3 wt%). The composition of the resulted CS‐PU hydrogel is DFPU 1.7 wt%, glycol chitosan 2 wt%, and 96.3 wt% water.

The functional groups of DFPU were confirmed by attenuated total reflectance Fourier transform infrared (ATR‐FTIR) spectroscopy. A peak representing the presence of aldehyde group was observed at 1380 cm^−1^ (C—H bending of aldehyde) (Figure S1A, Supporting Information). The X‐ray diffractometer (XRD) patterns for nonfunctionalized polyurethane (abbreviated as PU) and DFPU are displayed in Figure S1B (Supporting Information). The data indicated that nonfunctionalized PU was amorphous. After functionalization, the crystalline PCL peaks at 2θ = 21.21° and 23.51° showed up. The average molecular weight, the average hydrodynamic diameter, and the zeta potential of PU and DFPU are shown in Table S1 (Supporting Information). The zeta potentials of the PU NPs and the DFPU NPs were −57.2 ± 0.4 and −51.4 ± 0.9 mV, respectively. These values indicated good stability of the prepared dispersions. The average hydrodynamic diameter of PU NPs was 36.0 ± 0.6 nm and that of DFPU NPs was 39.5 ± 9.6 nm, respectively. The data for DFPU' are also demonstrated in Table S1 (Supporting Information). The images from transmission electron microscopy, displayed in **Figure** **2**, showed that the DFPU NPs had a spherical shape with a diameter of about 40 nm (Figure 2A), and after mixing with CS, transformed into irregular oval shape (Figure 2B). The small‐angle X‐ray scattering (SAXS) profiles for DFPU dispersions in different solid contents are shown in Figure 2C. When the solid content increased, the flat area (black arrow) in the q range 0.01 was more apparent, an influence from the structure factor of DFPU NPs. In Figure 2D, for the homogeneous mixture of DFPU and CS, the hump peak (black arrow) in the q range 0.01 became obvious after the sol–gel transition, indicating that DFPU NP deformed after crosslinking CS.

### 2.2 Optimization of the Composition for the CS‐PU Hydrogel (Contents of Main Chain and Crosslinker)

**Figure 3:**
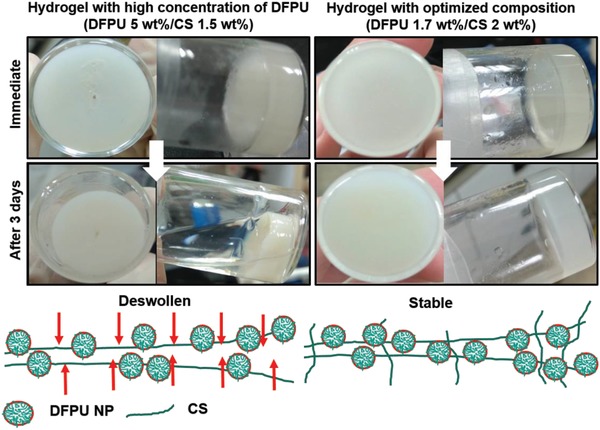
Optimization of the ratio of DFPU nanoparticulate crosslinker and glycol chitosan (DFPU 1.7 wt%/CS 2 wt%) to form stable CS‐PU hydrogel without deswelling. A higher ratio of DFPU (DFPU 5 wt%/CS 1.5 wt%) resulted in shrinkage and deswelling (dehydration) of the hydrogel in 3 d. Details of optimization are supplemented in Table S2 (Supporting Information).

The CS‐PU hydrogel was conveniently achieved by mixing DFPU and CS. To optimize and select the proper composition to form CS‐PU hydrogel with better properties, the amounts (concentrations) of DFPU and CS were adjusted as listed in Table S2 (Supporting Information). For the compositions DFPU 5 wt%/CS 1.5 wt%, DFPU 2.5 wt%/CS 1.5 wt%, and DFPU 2.3 wt%/CS 2 wt%, the hydrogel shrank and dehydrated after storage. For the composition DFPU 1.7 wt%/CS 2 wt%, the hydrogel remained stable (**Figure** **3**). For the composition DFPU 1 wt%/CS 2 wt%, a hydrogel could not form ever after 3 d. A saturated water content was achieved when the molar ratio of amine group (CS)/aldehyde group (DFPU) was 1:0.005. A higher proportion of DFPU (0.007) let to dehydration and a lower proportion of DFPU (0.003) failed to form network. These observations indicated that the ratio of the main chain CS and the crosslinker DFPU had a significant influence on the stability of the CS‐PU hydrogel. The composition DFPU 1.7 wt%/CS 2 wt% was selected to perform subsequent experiments.

### 2.3 Characteristics of the CS‐PU Self‐Healing Hydrogel

**Figure 4:**
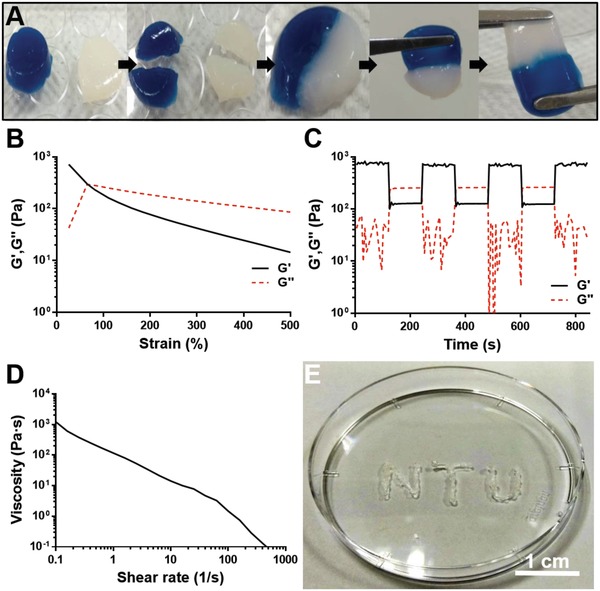
Characteristics of the CS‐PU self‐healing hydrogel (from DFPU 1.7 wt% and CS 2 wt%). A) Macroscopic hydrogel recovery process. B–D) Rheological properties of the hydrogel. E) Needle injectability. In (A), two circular samples were cut into half and then cross placed together for 5 h. After that, the healed sample was stretched by a pair of tweezers. In (B), the strain for the deconstruction was evaluated by the strain sweep (1–500% strain) experiment at 37 °C and 1 Hz. The gel to sol transition occurred when the strain was ≧ 80%. In (C), the damage‐healing properties of hydrogels were demonstrated by measurements under three cycles of the strain change (1% strain → 130% strain → 1% strain → …) at 37 °C and 1 Hz, and the CS‐PU hydrogel could restore its structure after high strain‐induced structural damage, i.e., with the self‐healing capability. In (D), the static shear viscosities of CS‐PU self‐healing hydrogel versus the shear rate at 37 °C. In (E), the CS‐PU self‐healing hydrogel could be injected through a 26‐gauge needle (260 µm internal diameter).

In **Figure** **4**A, two hydrogels with different colors were prepared and then cut into two semicircular pieces, and put together for observation of self‐healing at room temperature. One hour later, the scar at the damage site disappeared. The healed gels could support their own weight after healing and endure stretching without breaking at the cut/healed position.

The time‐dependent and frequency‐dependent viscoelasticity of the CS‐PU hydrogel at 37 °C are shown in Figure S2 (Supporting Information). The stabilized storage modulus (G′) of the hydrogel was ≈700 Pa at 37 °C and 1 Hz (Figure S2A, Supporting Information). There was no significant difference in the stiffness of the hydrogel in the frequency range of 1–100 rad s^−1^ (Figure S2B, Supporting Information). The strain‐dependent viscoelasticity of the hydrogel at 1 Hz 37 °C is shown in Figure 4B. The G′ values decreased as the dynamic strain increased over the range of 1 to 500%. The gel‐to‐sol transition occurred when the strain exceeded 80%. Finally, the damage‐healing cycles evaluated by successive step changes in the dynamic strain between 130% and 1% as demonstrated in Figure 4C. Upon the change to the higher strain (130%), the G′ of the hydrogel dropped from ≈700 Pa to ≈100 Pa and was lower than G″. Repeated damage‐healing experiments showed that the self‐healing hydrogel could fully restore their structure after multiple cycles. In Figure 4D, the static experiment revealed that the steady shear viscosity of the hydrogel decreased as the shear rate increased, which indicated that the CS‐PU hydrogel exhibited shear‐thinning property and suggested good injectability. Indeed, the CS‐PU hydrogel could be injected through a 26‐gauge (260 µm internal diameter) needle, as shown in Figure 4E.

### 2.4 Characteristics of the CS‐PU Cryogel

**Figure 5:**
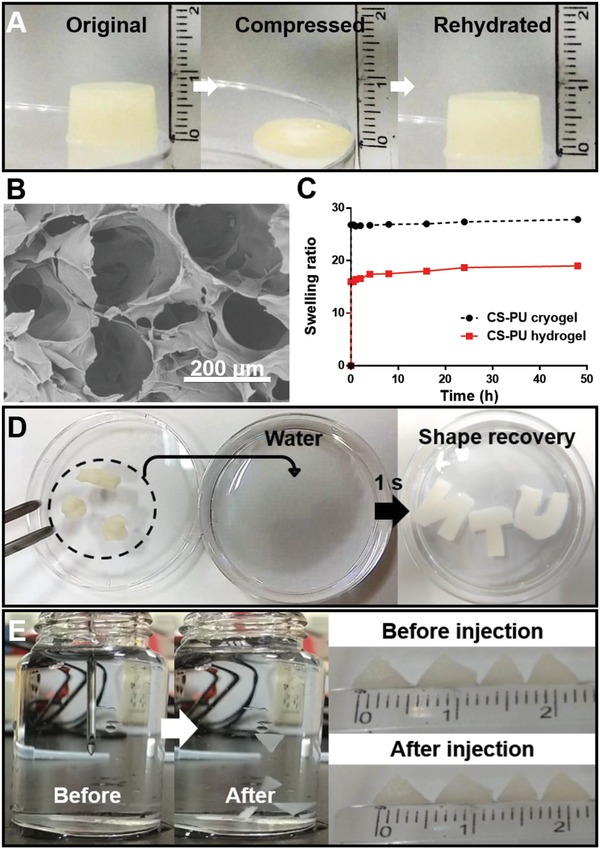
Characteristics of the CS‐PU cryogel (from DFPU 1.7 wt% and CS 2 wt%). A) The compressed cryogel could return to the original shape after rehydration. B) The SEM cross‐sectional image of CS‐PU cryogel showing interconnected macroporous network. C) Water swelling of the lyophilized hydrogels and cryogels, against the immersion time. D) Cryogels distorted by external force could return to the original shape in 1 s after immersion in water. E) The cryogel (length 4 mm, thickness 1 mm) could be injected by a conventional 18‐gauge needle (838 µm internal diameter) and recover the original shape after injection in water without being distorted.

CS solution was mixed with DFPU dispersion at −20 °C for overnight to obtain CS‐PU cryogel. The bulk composition of CS‐PU cryogel was the same as that of CS‐PU hydrogel (DFPU 1.7 wt%/CS 2 wt%). As displayed in **Figure** **5**A, the CS‐PU cryogel was compressed (1 mm height) and then recovered their initial shape (8 mm height) by rehydration. The compressibility was about 87.5%. The mechanical strength, swelling degree, and porosity of the cryogel are listed in Table S3 (Supporting Information). In addition to the high swelling ratio of 2730%, the CS‐PU cryogel absorbed water very fast, demonstrated in Movie S1 (Supporting Information). In Figure 5B, the image from scanning electron microscopy (SEM) showed interconnected macroporous (250 µm) network in the cross‐section of CS‐PU cryogel. Meanwhile, the freeze‐dried self‐healing hydrogel showed a different porous structure, i.e., the holes were smaller (100 µm) and fewer holes than those of the CS‐PU cryogel (Figure S3, Supporting Information). The profiles of water swelling for the lyophilized hydrogels and cryogels against the immersion time at 37 °C are shown in Figure 5C. It was apparent that cryogels were swollen to around 27‐fold of the dried mass in less than 1 min but CS‐PU hydrogel could only be swollen to around 16‐fold of the dried mass in 1 min (and 19‐fold after 48 h). The thermal stability of CS‐PU hydrogel and CS‐PU cryogel is shown in Figure S4A,B (Supporting Information). Derivative thermogravimetry (DTG) is a type of thermal analysis in which the rate of material weight changes upon heating is plotted against temperature. The DTG curves indicated that both CS‐PU hydrogel and CS‐PU cryogel were stable at high temperatures up to about 200 °C. Besides, the structure of CS‐PU cryogel was more heterogeneous than that of CS‐PU hydrogel.

Deformed CS‐PU cryogels had the ability to recover to the original shape within 1 s, as illustrated in Figure 5D and Movie S2 (Supporting Information). Moreover, the triangle‐shaped CS‐PU cryogels (5 mm side length, 1 mm thickness) could be squeezed through an 18‐gauge needle (838 µm inner diameter) and immediately return to their original geometry after injection, as illustrated in Figure 5E and Movie S3 (Supporting Information).

### 2.5 The Responsive Ability and In Vitro Degradation of CS‐PU Hydrogel and Cryogel

**Figure 6:**
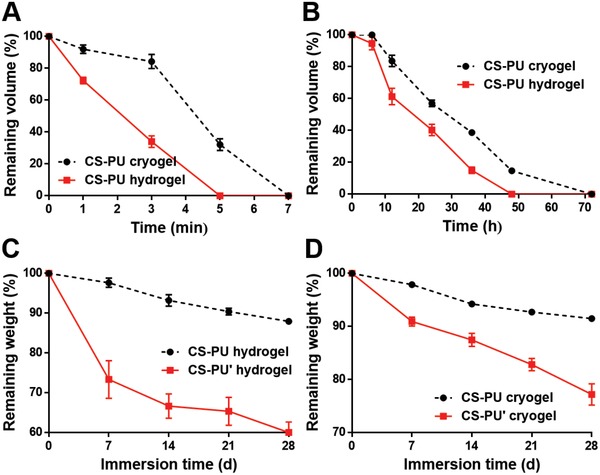
The responsive ability and in vitro degradation of cryogels and hydrogels (from DFPU 1.7 wt% and CS 2 wt%). The CS‐PU cryogel and CS‐PU hydrogel were soaked in A) acetic acid and B) aniline, where the remaining volume of the cryogel and hydrogel were measured and compared. The remaining volume 0% indicates dissolution. Meanwhile, in vitro degradation was conducted by immersion in PBS at 37 °C. C) The degradation of CS‐PU hydrogel was compared with that of CS‐PU' hydrogel, and D) the degradation of CS‐PU cryogel was compared with that of CS‐PU' cryogel to demonstrate the tunable degradation rate.

The pH and aniline responsiveness of CS‐PU hydrogel and cryogel is summarized in **Table** **1** and **Figure** **6**A,B. CS‐PU hydrogel and cryogel were fully liquefied after soaking in acetic acid for 5 and 7 min, respectively (Figure 6A). In contrast, CS‐PU hydrogel and cryogel were fully liquefied after soaking in aniline for 48 and 72 h, respectively (Figure 6B). In vitro degradation profiles in 37 °C phosphate buffered saline (PBS) are demonstrated in Figure 6C,D. Gels prepared from CS with DFPU' crosslinker were named as CS‐PU' gels. After 28 d CS‐PU hydrogel remained 87.8% by weight, while CS‐PU' hydrogel remained 60.0% by weight (Figure 6C). Meanwhile, CS‐PU cryogel remained 91.5% by weight, and CS‐PU' cryogel remained 77.1% by weight after 28 d (Figure 6D). The comparative degradation profiles of PU films are also shown in Figure S5 (Supporting Information). In addition, the gel fraction for PU films, CS‐PU hydrogels, and cryogels is shown in Figure S6 (Supporting Information). No obvious difference in the gel fraction was observed among CS‐PU hydrogel, CS‐PU' hydrogel, CS‐PU cryogel, and CS‐PU' cryogel. Taken together, these data suggested that by changing the soft segment used in the synthesis of the crosslinker, the degradation rates of the resulted hydrogels and cryogels could be adjusted.

**Table 1:** Responsive ability of CS‐PU hydrogel and CS‐PU cryogel

Materials	Exposed to	Outcome
CS‐PU hydrogel	1 mL 97% acetic acid (pH < 1)	Liquefied in 5 min
	1 mL aniline (pH ≈ 10)	Liquefied after 48 h
CS‐PU cryogel	1 mL 97% acetic acid (pH < 1)	Liquefied in 7 min
	1 mL aniline (pH ≈ 10)	Liquefied after 72 h

### 2.6 Cell Survival and Proliferation in the CS‐PU Hydrogel and CS‐PU Cryogel

**Figure 7:**
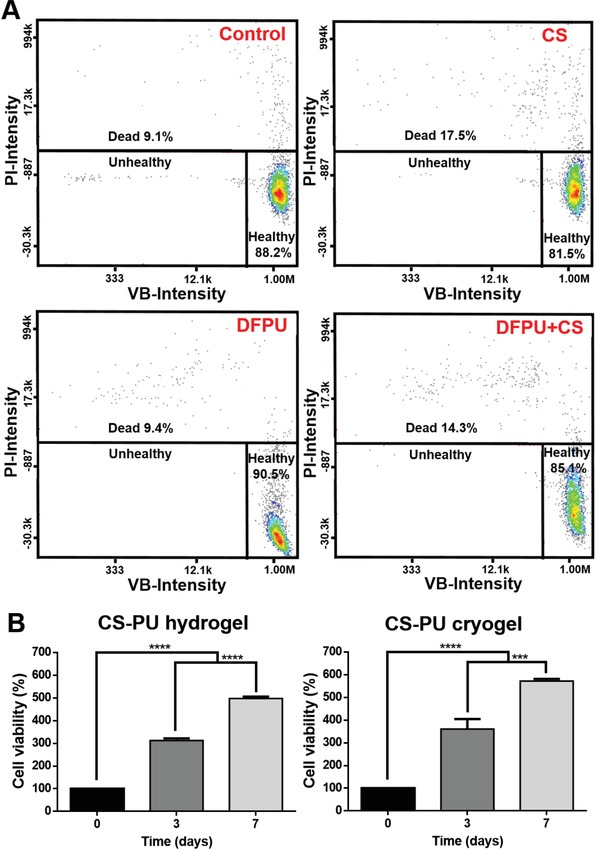
Cell survival and proliferation in the CS‐PU hydrogel and the CS‐PU cryogel. A) The vitality of NSCs determined by the VB‐48 assay. The data of control group were obtained from cells in the culture medium. B) The viability and proliferation of NSCs embedded in the CS‐PU hydrogel and CS‐PU cryogel determined by the CCK‐8 assay. The cell viability value (%) was calculated from optical density after deduction from the blank control (i.e., the hydrogel or the cryogel without cells) and normalized to that of initial cells. ***p < 0.001 and **** *p* < 0.0001 among the indicated group.

The immediate viability of neural stem cells (NSCs) in CS‐PU hydrogel evaluated by the VB‐48 assay is shown in **Figure** **7**A. The cell viabilities in the groups of medium, CS, DFPU, and DFPU+CS were 88.2%, 81.5%, 90.5%, and 85.1%, respectively. There was no significant difference in the cell viability among the groups. Meanwhile, cell survival in longer term was assessed by the cell growth over 7 d of culture. The data are shown in Figure 7B. After 3 d, the amounts of cells were ≈312% and ≈360% in CS‐PU hydrogels and CS‐PU cryogels, respectively, compared to the initial values. After 7 d, the amounts of cells were ≈497% and ≈571% in CS‐PU hydrogels and CS‐PU cryogels, respectively.

### 2.7 Biocompatibility by Rat Subcutaneous Implantation

**Figure 8:**
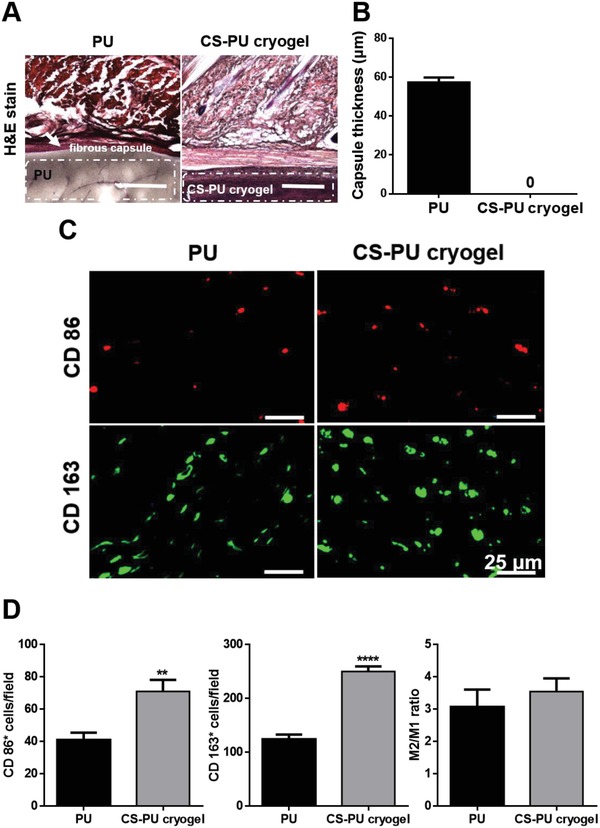
Foreign body reaction of CS‐PU cryogels after rat subcutaneous implantation. A) Histology of H&E‐stained sections after implantation for 14 d. The scale bar represents 500 µm. B) The extent of foreign body reaction could be revealed by the thickness of the fibrous capsule (white arrows) based on the histology. C) Immunofluorescent images (marker protein expression) of macrophages, stained by the mouse monoclonal anti‐CD86 antibody for M1 macrophages (red), or mouse monoclonal anti‐CD163 antibody for M2 macrophages (green). D) Quantification of M1 macrophage and M2 macrophage populations. Results are expressed as mean ± SD, *N* = 3. **p < 0.01, and **** *p* < 0.0001 among the indicated groups. PU (nonfunctionalized) films were used as the control.

The foreign body reaction was evaluated by histological staining of the explanted samples and the result is shown in **Figure** **8**A. Mild inflammation at the border of CS‐PU cryogel was observed after two weeks with the presence of inflammatory cells. In Figure 8B, PU (nonfunctionalized) was used as the control, which showed a fibrous capsule of 57.3 um thickness. CS‐PU cryogel did not show any fibrous capsule. In addition, immunofluorescence staining was performed to obtain the population ratio of M1 macrophages to M2 macrophages, as shown in Figure 8C,D. There was no significant difference in the M2/M1 ratio between CS‐PU cryogel and PU film. Both groups had an M2/M1 ratio of about 3, higher than that (about 0.5) reported for polylactide.^20^

## 3 Discussion

Biodegradable crosslinkers in the form of polyurethane nanoparticles were successfully synthesized by a green water‐based process. This type of crosslinker for producing dynamic Schiff bonding is rarely reported. According to the dynamic light scattering (DLS) measurement, the DFPU NPs were stably suspended in water. Furthermore, ATR‐FTIR results revealed that PU was successfully modified by glyoxal with aldehyde groups. Meanwhile, XRD patterns of PU films demonstrated that the modification induced crystallinity of the PCL segment was induced after modification. In addition, the morphology of DFPU was investigated by SAXS and observed by transmission electron microscopy (TEM). The TEM image of DFPU NPs showed the NPs in spherical shape. After mixing with CS, DFPU NPs gradually transformed into irregular oval shape gradually. The deformation of DFPU NPs was probably caused by the different reaction rates of aldehyde groups and amine groups during the crosslinking process.

Optimal synthetic conditions exert a significant influence on the properties of the product.^21^ The formation of the hydrogel was optimized with the procedures described below. First of all, the undiluted DFPU (28 wt%) was mixed with CS (3 wt%). In observations of the properties of CS‐PU hydrogel, the hydrogels underwent syneresis after 24 h. According to the literature, the properties of swelling and syneresis were correlated to the effective crosslinking density described by the polymer network theory.^22^ Therefore, the crosslinker DFPU dispersion was prepared in various concentrations to optimize the composition of the hydrogel. After the formation of hydrogel, the crosslinking reaction kept going, which increased the crosslinking density. When the crosslinking density was too high, the structure of the hydrogel started to shrink, causing dehydration. The shape of the hydrogel could be maintained for a longer period of time when the proportion of the main chain (CS) in the hydrogel increased. The hypothetical mechanism for the formation of deswelling hydrogels and stable hydrogels is proposed in Figure 3. In addition, it was unable to form a hydrogel when the concentration of DFPU was too low. These results indicated that the hydrogel was stable and water‐saturated when the ratio of crosslinker to main chain was optimized. Considering the stability of CS‐PU hydrogel, the composition DFPU 1.7 wt%/CS 2 wt% was selected for major experiments.

The CS‐PU hydrogel was prepared by Schiff reaction from the aldehyde group of DFPU and the amine group of CS. As mentioned previously, dynamic Schiff base could lead to the self‐healing ability of CS‐PU hydrogel. The healed gel could endure stretching without breaking at the cut/healed position and the rheometer was utilized for the further analysis of self‐healing properties of hydrogel. We found that the mechanical strength of hydrogel was close to the initial state after multiple cycles of damage‐healing processes, indicating the quick recovery of the inner network of CS‐PU hydrogel. CS‐PU hydrogel not only showed favorable self‐healing ability but revealed strong shear thinning behavior. To be more specific, CS‐PU hydrogel could be thinned into liquid form under the high shear rates and recovered back into gel rapidly after removal of shear stress. With such characteristics, the CS‐PU hydrogel was not only suitable for subcutaneous injection through a 26‐gauge needle, but may also have potential for future applications in 3D printing.

We unexpectedly discovered in our study that the mixture of DFPU and CS could form cryogel after freezing at −20 °C in our study. Self‐healing hydrogels and cryogels made of the same main chain and the crosslinker has not been reported so far. According to the literature, the formation rate of crosslinking network and ice crystal exerted a significant influence on the generation of a cryogel.^23^ Therefore, we assumed that the DFPU crosslinker played an important role on forming CS‐PU cryogels. The spherical crosslinker resulted in the formation of unique network possibly because each aldehyde group on the NPs exhibited different steric hindrance on the main chain. When the mixture of CS and DFPU was frozen, the ice crystals in the mixture grew until they met the facets of other crystals. DFPU crosslinked the chains of CS tightly around the ice crystals. After thawing the ice crystals, interconnected pores were generated inside the gel. Because of the interconnected macropores (250 µm) in the cryogel, the CS‐PU cryogel had higher swelling ratio and swelling rate than that of freeze‐dried CS‐PU hydrogel. In addition, CS‐PU cryogel (5 mm side length, 1 mm thickness) could be squeezed through an 18‐gauge needle (838 µm inner diameter) without shape loss or gross damage. In our study, the compressibility of target cryogel was consistent with its high degree of porosity. The result indicated that the CS‐PU cryogel could be compressed to almost its minimum volume and injected through a needle. The CS‐PU cryogel regained its geometry and architecture after being injected. According to the literature, cryogels were suitable as carriers for minimally invasive delivery of adherent cells with effective protection from compression forces during injection.^24^ Compared with traditional procedures, CS‐PU cryogels were able to be injected to a specific location without invasive surgery, which could decrease scarring, lessen the risk of infection, and reduce recovery process.

CS‐PU hydrogel and cryogel developed here were sensitive to acetic acid and aniline. On the basis of the literature, Schiff bases were often sensitive to hydrolysis under acidic conditions,^25^ which accounted for the liquefaction of both CS‐PU hydrogels and cryogels under acidic conditions. In addition, when treated with aniline, CS‐PU hydrogels and cryogels were also liquefied. Aniline with monoamine group could compete with CS to crosslink DFPU, leading the destruction of 3D network.^12^ Besides chitosan, many biomacromolecules containing amine groups are expected to form gels through the crosslinking of DFPU based on Schiff base. Meanwhile, the gels with multiresponsive properties may have interests in drug release.

Degradation of the hydrogels after implantation is important in tissue engineering. The degradation rates should be adjusted in order to suit for various tissues. Gels with tunable degradation rates are highly demanded. Hsieh et al. prepared a gel slowly degraded to provide better support for vasculogenesis in vivo.[qv: 13b] Hsu et al. developed green/water‐based, biodegradable polyurethanes of which the degradation rate could be altered by the types of the component oligodiols.^26^ In the present study, two of the water‐based polyurethanes (DFPU and DFPU') were functionalized as crosslinkers. It is reasonable to assume that the degradation rates of CS‐PU hydrogels and cryogels prepared with different types of crosslinkers remained to be tunable. In fact, CS‐PU degraded slower than CS‐PU'. We speculated that the soft segments of the NP crosslinker developed here may be further changed to obtain hydrogels and cryogels with desired properties.

Analyses of cell viability and cell growth were performed on CS‐PU hydrogels and cryogels in vitro to evaluate the tissue engineering applications. The results indicated that no significant difference was observed in the immediate cell viability among the control group (culture medium), DFPU, CS, and DFPU+CS. Meanwhile, significant cell proliferation was confirmed after 7 d of cell culture in CS‐PU hydrogel and cryogel. These data supported the cytocompatibility of DFPU crosslinker, CS‐PU hydrogel, and CS‐PU cryogel. The hydrogel as negative control group was prepared by mixing glyoxal and CS (Figure S7, Supporting Information). After 3 and 7 d, the amounts of cells in negative control groups were lower than those in the experimental CS‐PU hydrogel groups, indicating that the crosslinker DFPU was superior to the conventional crosslinker glyoxal in the biocompatibility. In terms of the mechanical properties, the literature indicated that the matrix of 0.1–10 kPa stiffness could offer a favorable environment for neural cells.^27^ During the long‐term culture, we found that the degradation rates of CS‐PU hydrogel and cryogel both increased, probably because of the pH change in the microenvironment around the samples. In addition, amine‐containing molecules from the metabolism of cells could also influence the degradation rates of the gel. These conditions may contribute to the degradation of CS‐PU hydrogel and cryogel in a biological environment.

Rat implantation showed that the CS‐PU cryogel completely degraded after 28 d (Figure S8, Supporting Information), possibly because the multiple responsiveness of CS‐PU cryogel accelerated the biodegradation rates of the gel. Moreover, rat implantation at 14 d revealed mild inflammation at the border of CS‐PU cryogel. According to the literature, the M2 macrophages play an essential role in tissue remodeling and suppression of inflammatory immune reactions.^28^ In the present study, CS‐PU cryogel as well as PU film (control group) recruited less M1 macrophages and more M2 macrophages than PLA films. These data indicated that CS‐PU cryogel was superior to the conventional PLA in biocompatibility. Polyurethane nanoparticles and films were reported to inhibit the macrophage polarization toward the M1 phenotype.^20^ The good individual biocompatibility of PU and CS may explain the low immune reaction of the CS‐PU cryogel.

In summary, we developed a novel biodegradable NP crosslinker which could be used to fabricate CS‐PU self‐healing hydrogels and cryogels with good cytocompatibility, biocompatibility, injectability, and multiresponsiveness. Moreover, the degradation rates of target gels could be further fine‐tuned by changing the soft segment of the crosslinker. The CS‐PU gels are promising biomedical materials and may have good potential in tissue engineering applications.

## 4 Conclusion

Novel waterborne and biodegradable PU NP crosslinker dispersions with aldehyde groups were synthesized and characterized in this study. CS‐PU self‐healing hydrogels and cryogels were formed from the same ingredients by Schiff reaction between DFPU and CS under room temperature and −20 °C, respectively. These gels were found to be sensitive to pH values and amine‐containing molecules owing to the property of Schiff bases. The degradation rates of gels could be adjusted by the types of the component oligodiols in DFPU. Both CS‐PU self‐healing hydrogels and cryogels were injectable (26‐gauge and 18‐gauge needles, respectively) and demonstrated good cell proliferation (497% and 571% after 7 d, respectively). CS‐PU self‐healing hydrogels had excellent self‐healing properties that fully (≈100%) recovered after damage. CS‐PU cryogels exhibited great water absorption (≈2730%), compressibility (eightfold), and the ability of shape recovery. These materials showed low immune responses in rat 14‐d implantation. Therefore, DFPU crosslinkers and CS‐PU gels are promising new materials for biomedical applications.

## 5 Experimental Section

*Synthesis of DFPU Crosslinkers*: DFPU was synthesized as described earlier. PCL diol was purchased from Sigma (USA). PEBA diol was supplied from Greco (Taiwan). IPDI, DMPA, and EDA were acquired from Acros (USA), Sigma (USA), and Tedia (USA), respectively. MEK and TEA were both obtained from J.T. Baker (USA) and used as received. Glyoxal was purchased from Alfa Aesar (USA). Nonfunctionalized PU, to contrast DFPU, was synthesized from a waterborne process previously developed.^29^ The process was similar to that of DFPU except that no glyoxal was added.

*Characterization of DFPU Crosslinkers*: The waterborne DFPU synthesized was in the nanoparticle form. The hydrodynamic diameter (*D*_h_) and the zeta potential of the NPs in dispersion (3000 ppm) were measured by a nanoparticle analyzer (Delsa Nano, Beckman Counter) involving the principle of dynamic/electrophoretic light scattering. The morphology of the DFPU NPs in dispersion was examined by TEM (Hitachi H‐7100, Japan). The DFPU dispersion was diluted with distilled water to a concentration of 5000 ppm. The diluted DFPU dispersion was dropped on the copper grid for 2 min and removed of excess DFPU using a filter paper. After that, the phosphotungstic acid was dropped on the copper grid for 30 s before observation. The DFPU dispersions in various solid contents (5–30 wt%) were investigated by SAXS at the beamline 23A of the National Synchrotron Radiation Research Center (NSRRC) at Hsinchu, Taiwan.

DFPU dispersions were cast into films and characterized using an ATR‐FTIR spectrometer (Spectrum 100 model, Perkin Elmer). PU films cast from nonfunctionalized PU dispersion were also analyzed for comparison.

The size distribution (polydispersity index, PDI) of DFPU and the molecular weight (Mw and Mn) were obtained by the gel permeation chromatography (GPC, JASCO, Japan) coupled with an RI (RI‐930) detector using *N*,*N*‐dimethylacetamide (DMAc, Tedia) as the eluent.

The diffraction peaks of DFPU were investigated using an XRD (X'Pert, PANalytical, Netherland). The pyrolytic temperature of DFPU was obtained using the thermogravimetric analyzer (TGA, Q50, TA, USA) at a heating rate of 10 °C min^−1^ under N_2_.

*Preparation of CS‐PU Self‐Healing Hydrogels and CS‐PU Cryogels*: The water‐soluble CS (Wako, Japan) was crosslinked by DFPU to prepare CS‐PU self‐healing hydrogels and CS‐PU cryogels. Glycol chitosan (CS) powder was dissolved in deionized water and vortexed in 3 wt% concentration. The crosslinker DFPU dispersion was prepared in various concentrations (10, 7, 5, and 3 wt%) to optimize the composition of the hydrogel. CS solution was mixed with DFPU dispersion by vortex under room temperature to form a homogenous mixture, which were CS‐PU self‐healing hydrogels.

The cryogel composition was selected with the ratio of optimized ingredients. To prepare the cryogel, CS solution was mixed with DFPU dispersion by vortex under room temperature and then immediately placed in a freezer set at −20 °C for overnight.

*Characterization and Rheological Evaluation of CS‐PU Self‐Healing Hydrogels*: The morphology of the DFPU NP crosslinked CS was examined by a TEM (Hitachi H‐7100, Japan). The DFPU dispersion mixed with CS solution was diluted with distilled water to a concentration of 5000 ppm. Sample preparation followed that described previously. The microstructure of DFPU mixed with CS was also examined by the SAXS at the beamline 23A of the NSRRC.

For visualization of the self‐healing property of the CS‐PU hydrogel, two hydrogel disks were separately prepared. Trypan blue was added in one hydrogel disk to make the color different. Each of the white and blue hydrogels were cut into two pieces and put two semicircles of different colors together to test if they formed a united disc.

Rheological properties of the CS‐PU self‐healing hydrogel was examined by a rheometer (Rheometric RS5, TA) at 37 °C. The storage modulus and loss modulus (G' and G'') were determined against time at a constant frequency of 1 Hz (6.28 rad s^−1^) and 1% strain. After that, the frequency sweep was obtained at a constant strain of 1% in the angular frequency range (1−100 rad s^−1^). The dynamic strain sweep was evaluated at a frequency of 1 Hz from 0.1% to 500% strain. The shear thinning property was analyzed by the steady shear experiment, where the viscosity was measured against the shear rate. For quantitative evaluation of the self‐healing process, G' and G'' at the constant frequency of 1 Hz were measured by damage‐healing cycles at the high strain (130%) for damage and at the low strain (1%) for healing.

*Characterization and Compression Property of CS‐PU Cryogels*: The porous structure of the CS‐PU cryogel (cross‐section) was examined by SEM (Hitachi TM3000, Japan) operated in 3 kV. The porosity of the CS‐PU cryogel was measured by immersion in ethanol. The percent porosity was calculated by the equation(*W*_w_ − *W*_d_)/*ρV* × 100%, where *W*_w_ was the wet weight of CS‐PU cryogel after immersion in ethanol, *W*_d_ was the dry weight of CS‐PU cryogel before immersion, ρ was the density of ethanol, and *V* was the volume of the CS‐PU cryogel.

The water swelling ratio of the cryogel was measured against time at 37 °C. Pre‐weighed dry cryogels (*W*_d_) were immersed in deionized water. The swollen weight (*W*_w_) of the cryogels was surveyed after removal of excess water from the surface. The swelling ratio of the cryogel was calculated by the equation (*W*_w_ − *W*_d_)/ *W*_d_ × 100%.

The dynamic compression modulus of the cryogel was evaluated with a dynamic mechanical analyzer (DMA, Q800, TA, USA) at a constant frequency of 1 Hz and 0.1% compression strain at 37 ^ο^C.

The ability of CS‐PU cryogel to flow through a conventional‐gauge needle and to regain the original shape after injection was examined. Triangle‐shaped CS‐PU cryogels were suspended in 1 mL of water and injected by an 18‐gauge needle.

*Responsiveness and In Vitro Degradation of CS‐PU Hydrogel and CS‐PU Cryogel*: The environmental responsiveness of the CS‐PU hydrogel and cryogel was tested by immersion of the sample (0.9 mL) in 1 mL acetic acid and aniline, respectively. The volume of samples remained in each solvent at each time point (*V*) was measured. In vitro degradation of CS‐PU hydrogel and cryogel was evaluated by immersion in PBS under 37 °C. Degradation of the hydrogel and cryogel prepared from CS using DFPU' instead of DFPU was also evaluated for comparison. Before the experiment, CS‐PU gels and CS‐PU' gels were lyophilized for 48 h and weighed (*W*_i_). Samples were washed with deionized water, freeze‐dried, and weighed (*W*_f_) after 7, 14, 21, and 28 d. The remaining weight (%) of samples was obtained by the equation *W*_f_/*W*_i_ × 100%.

The degree of chemical crosslinking for the DFPU films, CS‐PU gels, and CS‐PU' gels was estimated by gel fraction. The samples were lyophilized for 24 h and weighed (*W*_i_). Samples were immersed in MEK for 24 h. They were freeze‐dried for 24 h and weight (*W*_f_). The gel fraction (%) for each sample was obtained from the equation (*W*_f_/*W*_i_) × 100%.

*Cell Culture and Cell Viability Analysis*: NSCs were obtained from adult mouse brain. NSCs were cultured in the medium containing 1:1 Ham's F12 (Gibco) and HG‐DMEM supplemented with 10% fetal bovine serum (FBS) and 400 µg mL^−1^ G418 (Invitrogen). Cell viability after exposure to various ingredients (medium, DFPU, CS and DFPU+CS) for 10 min was analyzed by the VitaBright‐48 (VB‐48) assay. The viability of cells was determined by staining with VitaBright‐48 (VB‐48, blue) and propidium iodide (PI, red) solution. VB‐48‐positive and PI‐positive cells each represented viable and dead cells, and the respective population was quantified by the Nucleo‐Counter NC‐3000. Long‐term cell proliferation in the CS‐PU hydrogel or CS‐PU cryogel up to 7 d was obtained by the cell counting kit‐8 (CCK‐8, Sigma‐Aldrich) assay. The cell viability value (%) was calculated from optical density after deduction from the blank control (i.e., the hydrogel or the cryogel without cells) and normalized to that of initial cells.

*Rat Subcutaneous Implantation*: The CS‐PU cryogel and PU film (as the control) of ≈1 cm × 1 cm were inserted into the subcutaneous sites of the adult Sprague‐Dawley rats. The samples were removed after implantation for two weeks. The samples with surrounding tissues were fixed in formalin. The thickness of the fibrous capsule was obtained by examination under the optical microscope after hematoxylin and eosin (H&E) staining. The repair and the inflammatory response in vivo were investigated by immunofluorescence staining of tissue sections. The tissue sections were treated with mouse anti‐CD86 antibody (Bio‐Rad, USA) for staining of M1 macrophages, or mouse anti‐CD163 antibody (Bio‐Rad, USA) for staining of M2 macrophages at 4 °C overnight. After that, the tissue sections were incubated with secondary antibodies at room temperature for 1 h. The number of fluorescent cells was counted under a fluorescence microscope (Nikon, eclipse 80i, Japan). All procedures were approved by the Animal Care and Use Committee (NTU105‐EL‐00029).

*Statistical Analysis*: All the experimental data were independently confirmed for three times. Statistical analysis was represented by the Student *t*‐test. Results were considered statistically significant when *p* values were < 0.05.

## Conflict of Interest

The authors declare no conflict of interest.

## Supporting information

Supporting Information

Supplemental Video 1

Supplemental Video 2

Supplemental Video 3
