# Supplementary material for: Self‐Healing Hydrogels and Cryogels from Biodegradable Polyurethane Nanoparticle Crosslinked Chitosan
Source: Adv Sci (Weinh). 2019 Nov 11;7(3):1901388. doi: 10.1002/advs.201901388 (PMC7001655; doi:10.1002/advs.201901388)
Supplement: Supplementary file 1 — Supporting Information [file ADVS-7-1901388-s001.pdf]

Copyright WILEY-VCH Verlag GmbH & Co. KGaA, 69469 Weinheim, Germany, 2019.

ADVANCED SCIENCE
Open Access

Supporting Information

for *Adv. Sci.,* DOI: 10.1002/advs.201901388

Self-Healing Hydrogels and Cryogels from Biodegradable Polyurethane Nanoparticle Crosslinked Chitosan

*Tzu-Wei Lin and Shan-hui Hsu**

Supporting Information

Self-healing hydrogels and cryogels from biodegradable polyurethane nanoparticle crosslinked chitosan

*Tzu-Wei Lin, Shan-hui Hsu**

[*] Prof. S. Hsu, Tzu-Wei Lin
Institute of Polymer Science and Engineering, National Taiwan University, Taipei 10617,
Taiwan, R.O.C.
Phone: (886) 2-33665313
Fax: (886) 2-33665237
E-mail: shhsu@ntu.edu.tw

Supplemental Data

Table S1. The average molecular weight, polydispersity, zeta potential, and hydrodynamic diameter for difunctional polyurethanes and control (DFPU, DFPU', and PU) measured by GPC and dynamic light scattering at 25 °C, respectively. Difunctional polyurethanes DFPU and DFPU' had different soft segments in their chemical compositions. Non-functionalized polyurethane control was abbreviated as PU.

PU	Mn (*10^5 Da)	Mw (*10^5 Da)	Polydispersity (Mw/Mn)	Zeta potential (mV)	Hydrodynamic diameter (nm)
DFPU	0.90	1.33	1.5	-51.4±0.9	39.5±9.6
DFPU'	0.82	1.36	1.7	-45.55±0.8	32.8±7.6
PU	1.38	1.65	1.2	-57.2±0.4	36.0±0.6

Table S2. Optimization and selection of the composition (contents of main chain and crosslinker) for the CS-PU hydrogel.

Mixing process	Respective final content	Solid content	Molar ratio of –NH2/-CHO/H2O	Appearance of hydrogel after 3 days
DFPU 10 wt% 400 ul/ CS 3 wt% 400 ul	DFPU 5 wt%/ CS 1.5 wt%	6.5 wt%	1:0.019:907.810	Dehydration
DFPU 5 wt% 400 ul/ CS 3 wt% 400 ul	DFPU 2.5 wt%/ CS 1.5 wt%	4.0 wt%	1:0.010:932.083	Dehydration
DFPU 7 wt% 400 ul/ CS 3 wt% 800 ul	DFPU 2.3 wt%/ CS 2 wt%	4.3 wt%	1:0.007:696.635	Dehydration
DFPU 5 wt% 400 ul/ CS 3 wt% 800 ul	DFPU 1.7 wt%/ CS 2 wt%	3.7 wt%	1:0.005:701.489	Stable
DFPU 3 wt% 400 ul/ CS 3 wt% 800 ul	DFPU 1 wt%/ CS 2 wt%	3.0 wt%	1:0.003:706.344	Unable to form a hydrogel

Table S3. Summary of the mechanical, physical, and morphological properties of CS-PU cryogel (from DFPU 1.7 wt% and CS 2 wt%).

**Swelling ratio**	2730 ± 400%
**Porosity**	86.5 ± 1.6%
**Compression modulus**	5.8 ± 0.5 kPa

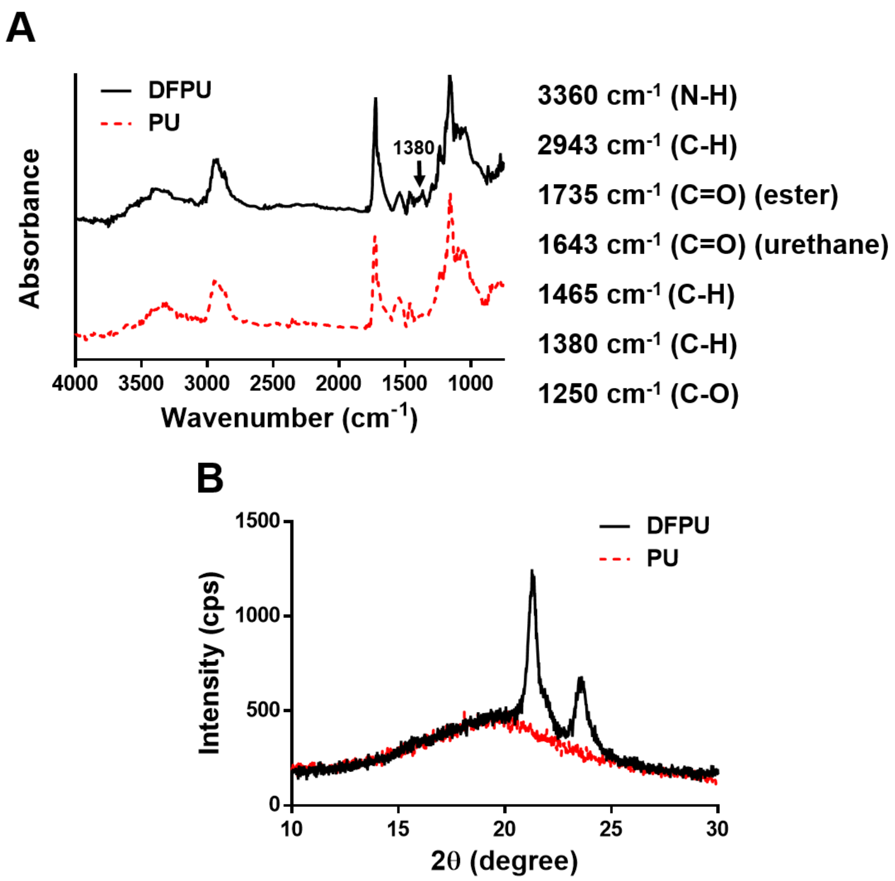

Figure S1. Characterization of DFPU by FT-IR spectroscopy and XRD. (A) PU was the control. DFPU was prepared by reacting polyurethane precursor with glyoxal. The characteristic peak of aldehyde group was observed (1380 cm^{-1} C-H bending) after the amine group of polyurethane reacted with the glyoxal by FT-IR spectroscopy. (B) The XRD profiles of DFPU and PU.

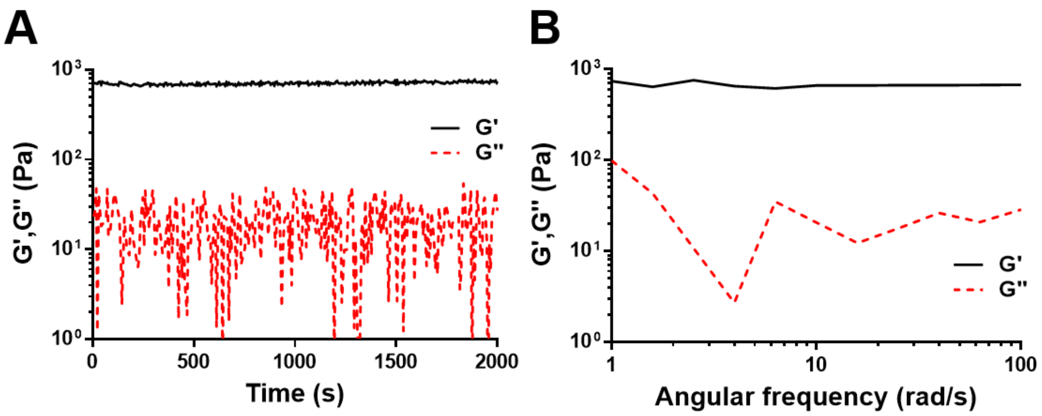

Figure S2. Additional rheological data for CS-PU self-healing hydrogel. (A) G’ and G’’ values were measured against time after mixing at 37 °C, 1 Hz, and 1% strain. (B) G’ and G’’ values were measured against frequency at 37 °C and 1% strain.

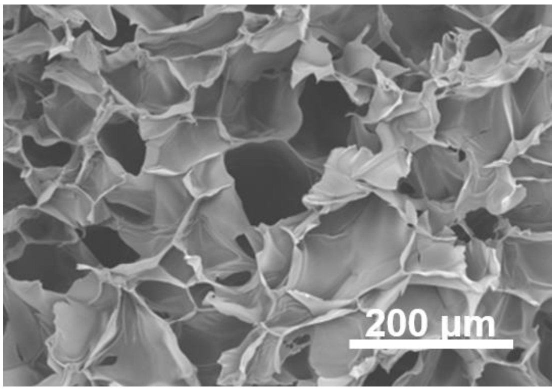

Figure S3. The SEM image for the cross-section of CS-PU hydrogel (freeze-dried sample).

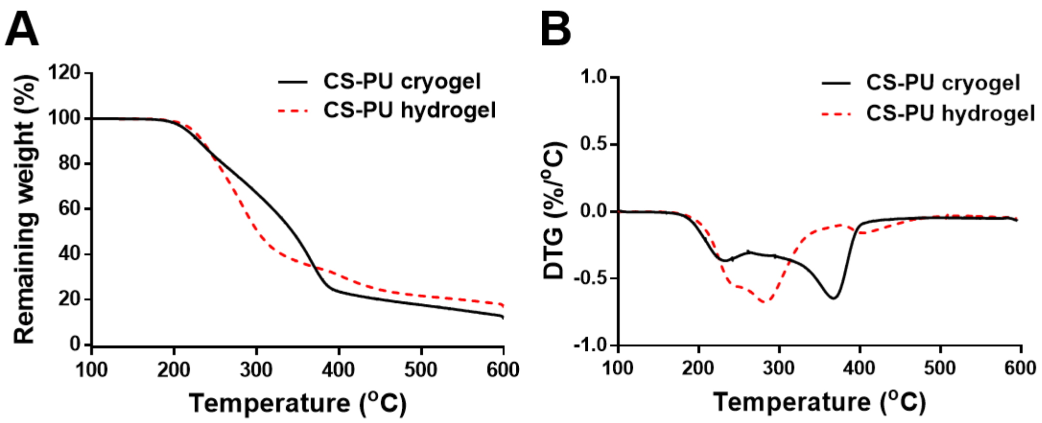

Figure S4. Thermal properties of the CS-PU cryogel and CS-PU hydrogel evaluated by TGA. (A) TGA profiles. (B) DTG curves. DTG is the first derivative of the TGA curve over temperature.

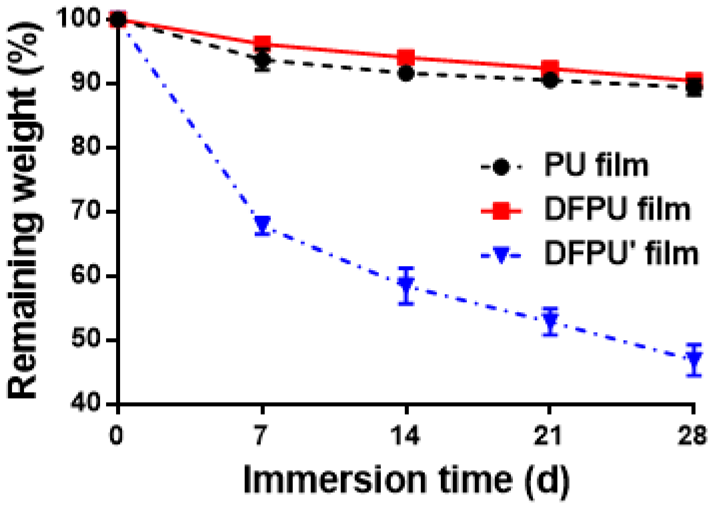

Figure S5. Comparative in vitro degradation profiles of the PU film, DFPU film, and DFPU' film immersed in PBS at 37 °C. The films were directly cast from the aqueous dispersion.

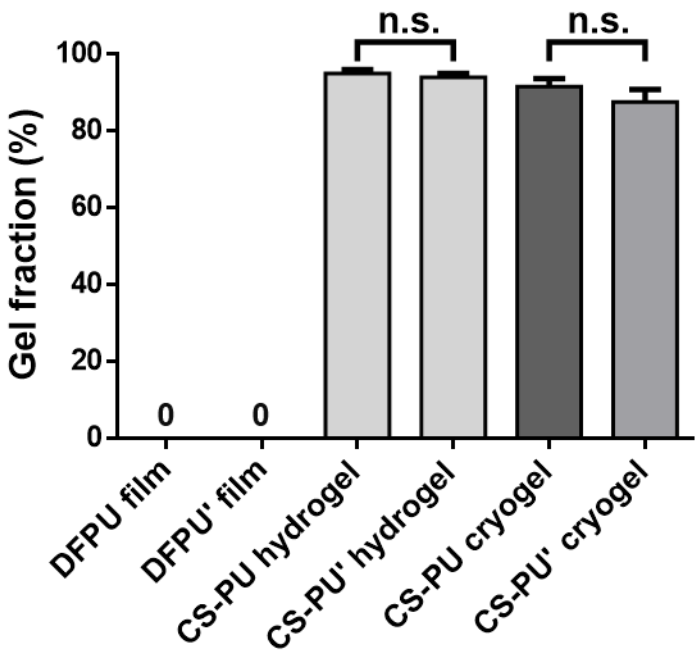

Figure S6. Degree of chemical crosslinking for PU films, CS-PU hydrogels, and cryogels, represented by gel fraction (%). Gels prepared using DFPU’ instead of DFPU as crosslinker were named as CS-PU’ gels.

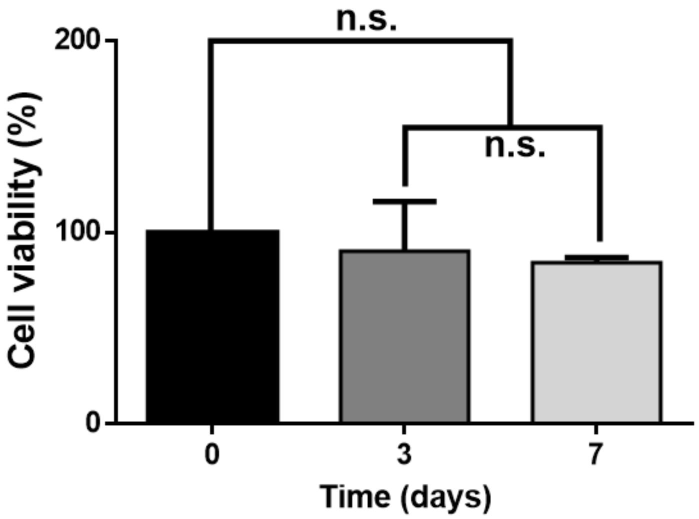

Figure S7. The viability and proliferation of NSCs embedded in the CS-glyoxal hydrogel (from glyoxal 0.007 wt% and CS 2 wt%) determined by the CCK-8 assay. The cell viability value (%) was calculated from optical density after deduction from the blank control (i.e. the hydrogel without cells) and normalized to that of initial cells. Based on the data, the CS-glyoxal hydrogel showed no proliferation of cells, and served as the negative control group.

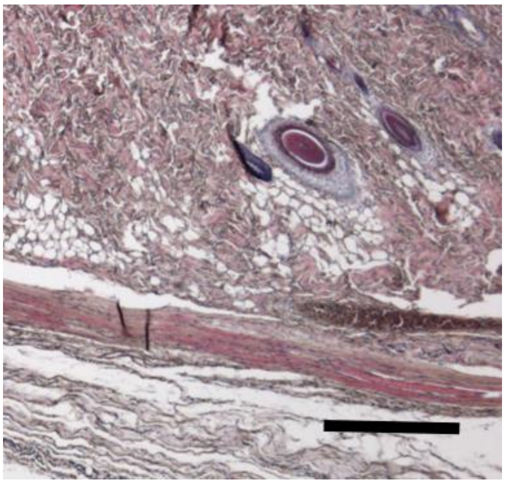

Figure S8. Histology of H&E-stained sections after implantation for 28 days. The scale bar represents 500 μm.

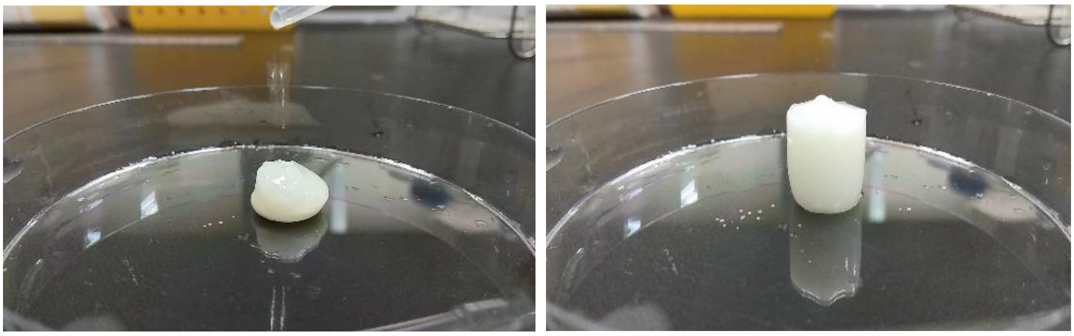

Movie S1. The compressed CS-PU cryogel can absorb the water droplet and swells immediately.

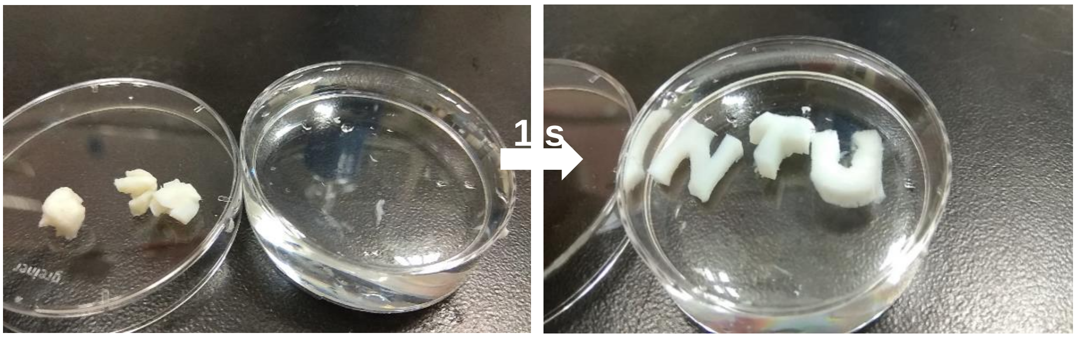

Movie S2. The pieces of cryogel returns to their original shapes after immersion into water.

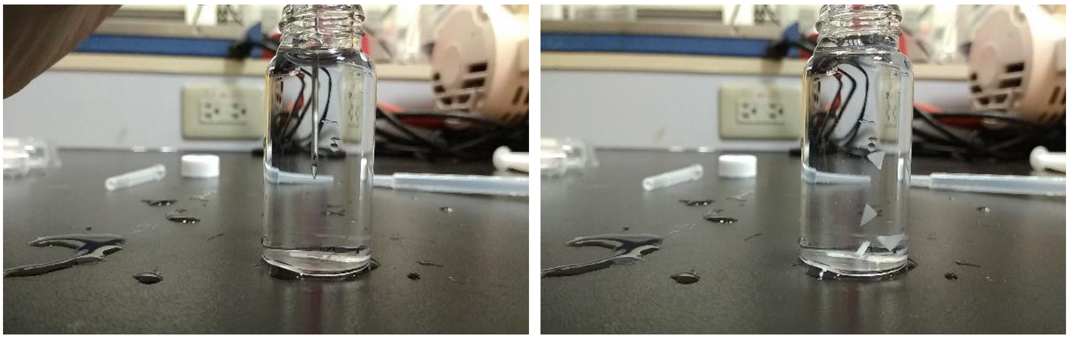

Movie S3. The pieces of cryogel (width 4 mm, thickness 1 mm) can be injected through a conventional 18-gauge needle (838 μm internal diameter) and retain their original shape.
